# Emergency Department Management of COVID-19 Suspected Patients. An International Perspective

**DOI:** 10.3389/ijph.2022.1604534

**Published:** 2022-04-29

**Authors:** Chady El Tawil, Mahmoud El Hussein, Nagi Souaiby, Mariana Helou

**Affiliations:** ^1^ Notre Dame Maritime Hospital, Byblos, Lebanon; ^2^ Gilbert and Rose-Mary Chagoury School of Medicine, Lebanese American University, Byblos, Lebanon; ^3^ Lebanese American University Medical Center Rizk Hospital, Beirut, Lebanon

**Keywords:** disaster, COVID-19, pandemic, global health, survey

## Abstract

**Objectives:** In December 2019, an invasive viral outbreak, the Corona Virus Disease 19 spread to the whole world. An international cross-sectional study was conducted to evaluate how healthcare workers in Emergency departments dealt with this pandemic.

**Methods:** A questionnaire was sent to 180 healthcare workers around the world during May and June of the year 2020.

**Results:** A total of 134 HCW from 23 countries responded with a majority of Emergency physicians (36.8%). The PCR testing is available in 72.9% of the hospitals. Different architectural strategies were used to isolate suspected cases in the Emergency department (ED). Half of the institutions would not allow visitors, while the other half, restricted visiting hours and the number of visitors. Triage for suspected patients relied in 82.8% on symptoms. Almost 98% of HCW used a combination of mask, gloves, gown and face shield. Around 65% of the HCW have a tendency to discharge more patients from the ED than what they were used to.

**Conclusion:** The COVID-19 pandemic made a major change within the emergency departments worldwide.

## Introduction

**FIGURE 1:**
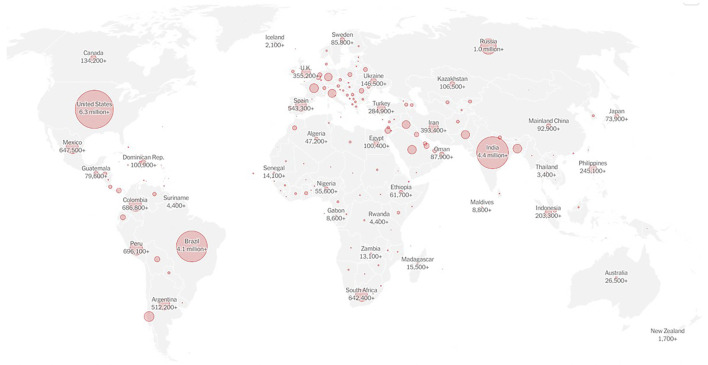
Coronavirus map: tracking the global outbreak, the New York times (New York, 2020). https://www.nytimes.com/interactive/2020/world/coronavirus-maps.html.

A viral outbreak spread in Wuhan, China in December 2019 [1]. The virus, labeled Corona Virus Disease 19 or COVID-19, spread worldwide rapidly and unexpectedly. By 11:46 a.m. in June 20, 2021, it had infected approximately 178 million people across the world, and the World Health Organization (WHO) had reported more than 3 million deaths [2]. On 11 March 2020, the WHO declared the COVID-19 outbreak as a pandemic (Figure 1) [3]. Even the wealthiest countries with the most developed healthcare systems could not contain this spread. The high transmission frequencies, the severity of the disease and the high mortality rates made the pandemic a real burden to societies [4]. Healthcare systems around the globe were overwhelmed, ranging from medical personnel to paramedics. Hospitals depleted their stocks of essential supplies and confronted significant scarcities with respect to Personal Protective Equipment’s (PPEs), respirators, and critical care beds [5, 6].

The ongoing pandemic is associated with decreased hospital admissions for cardiovascular and other acute care conditions [7, 8]. A study conducted in four large hospitals in Qatar compared the COVID-19 and pre COVID-19 periods, reporting a 20–43% drop in emergency department admissions, with the biggest decline observed in patients designated at the highest acuity [9]. The United States demonstrated similar results, registering a 35% decline in the ED visits in a single center, compared to the previous year [10].

As reported by a major hospital in Milan, hospitals worldwide were forced to deal with the pandemic, from expanding their space to accommodate more patients, to reorganizing and restructuring their EDs [11].

Triage criteria were initially limited to exposure or travel history and were then expanded to include any patient with suspected symptoms [11].

Apart from the heightened risks of getting infected, medical personnel began to show signs of burnout [5]. Many doctors and nurses were quarantined or contracted the disease because of the consistent and high exposure to a massive influx of patients, and numerous medical practitioners even died from the infection [4, 12]. More than 1000 doctors and nurses were included in the number of healthcare workers (HCW) who died due to COVID-19 infection [13, 14].

HCW used all available resources to face the pandemic and provide optimal care for their patients while also taking care to protect themselves. Masks, gloves, protective clothing, and goggles were the most common PPEs used. Guidelines were issued to ensure proper use of PPE in every healthcare environment in order to limit their use and avoid serious shortages [15, 16]. Some studies have confirmed the efficacy of full -body PPE [17]. In addition, the management of COVID-19 suspected patients and the directions for HCW protection varied between and within countries [10, 11, 15–18]. No reports have been found to document and compare discrete approaches used in EDs to manage COVID-19 suspected patients.

Therefore, the present study aimed to scrutinize the differences in management protocols for handling COVID-19 suspected patients. It also attempted to determine commonly implemented policies suitable enough to be generalized in other countries, which could potentially be applied during respiratory pandemics occurring in the future.

## Methods

An institutional review board (IRB) approval was obtained from the IRB committee of the Lebanese American University. This investigation was conducted in two stages. First, a pilot study was performed to evaluate the phrasing and the intelligibility of the 18 questions of the survey instrument. Fifteen residents handling COVID-19 patients at The Lebanese American University Medical Center in Beirut were asked to fill in and comment on the questionnaire. The remarks of the participants were evaluated, and the questions were edited accordingly. Supplementary Appendix S1 presents the final questionnaire. The questions were grouped into 2 parts: the first part relevant to demographic data: age, gender, country of work, specialty, hospital category, type of hospital. The second part related to COVID-19 management in the ER: PCR availability, triage, PPE use, ER architecture, management of patients, exposed staff, and visitors.

In the second phase, the questionnaire was sent *via* email to 250 participants worldwide, targeting HCW dealing with COVID-19 patients, including physicians, residents and certified nurses, working in adult EDs. The participants were located using social networking systems, with the stipulation that they should be HCW engaged in EDs and should be handling COVID-19 patients at the time of the study. Emails were sent over a period of 2 months between 1 May and 30 June 2020.

An online link was sent to ED heads, who were asked to forward the questionnaire to the HCW of their department. The questionnaire was anonymous and confidential. All HCW who were not physicians or nurses (e.g., paramedics, emergency technicians, and other practitioners) or the HCW that were not dealing with COVID-19 patients were excluded. The obtained data were analyzed and interpreted using SPSS statistics.

## Results

A total of 134 HCW (74%) returned the filled questionnaire by the end of the study period.

### Demographics

The participants were distributed as: 45.9% females and 54.1% males. The respondents belonged to 23 countries across six continents (Table 1): Asia (Lebanon, Kuwait, Pakistan and United Arab Emirates), Europe (France, Germany, Italy, Ireland, United Kingdom, Czech Republic, Luxemburg, Greece, Belgium, Croatia, Sweden), Africa (Algeria, Nigeria, Ethiopia, South Africa), North America (Canada, United States), South America (Argentina) and Oceania (Australia).

**TABLE 1:** The total number of healthcare workers and the number of each medical specialty in the different countries (Lebanon, 2020).

Medical specialty									
Country	Participants (N)	Emergency medicine	Internal medicine	Registered nurse	Surgery	General medicine	Anesthesia	Pulmonary/Intensive Care	Others
Algeria	15		1	1	4		3	1	5
Argentine	1							1	
Australia	1							1	
Lebanon	45	17	3	5	4	2	1	3	10
Belgium	2					1			1
Canada	3	3							
Czech Republic	1							1	
Croatia	1	1							
Deutschland	2		2						
Ethiopia	1	1							
France	15	6		1		1	1		6
Germany	4	1	1	1		1			
Greece	1	1							
Ireland	4	2		2					
Italy	2	2							
Kuwait	4			2		1	1		
Luxembourg	1						1		
Nigeria	1					1			
Pakistan	1					1			
South Africa	1								1
Sweden	4	3		1					
United Arab Emirates	3	2				1			
United Kingdom	5	3			2				
United States	16	8	4		1			1	2
Total Number (Percentage)	134	50 (37%)	11 (8%)	13 (10%)	11 (8%)	9 (7%)	7 (5%)	8 (6%)	25 (19%)

The majority of the participants were Emergency Physicians (36.8%), as distinguished from other physicians (53.4%). Nurses represented only 9.8% of the respondents (Table 1). Most participants worked at university hospitals and private hospitals (58.4% and 57.8%, respectively).

### Hospitals Resources

Polymerase chain reaction (PCR) testing for COVID-19 was available in 72.9% of the hospitals represented by the respondents.

The management of exposed HCW differed worldwide. Most institutions (65.4%) asked their exposed staff to stay at home and wait for the PCR test result, which was scheduled a few days after the exposure. Staff from other departments would cover the unit in the meantime. If the PCR result was negative and the HCW remain asymptomatic, they were allowed to resume work. Other institutions (11%) imposed a full 14 days quarantine on exposed staff members without requiring them to take a PCR test. Some hospitals (18.6%) also allowed staff members to continue working with full protective measures if they were asymptomatic. A few institutions (5%) implemented more individualized protocols.

In terms of allowing visitors, 48.9% of the institutions did not permit hospital visits at any time, while the remaining 51.1% demonstrated fewer restrictions, imposing strict visiting hours and limiting the number of visitors.

### The Management of COVID-19 Suspected Cases

**FIGURE 2:**
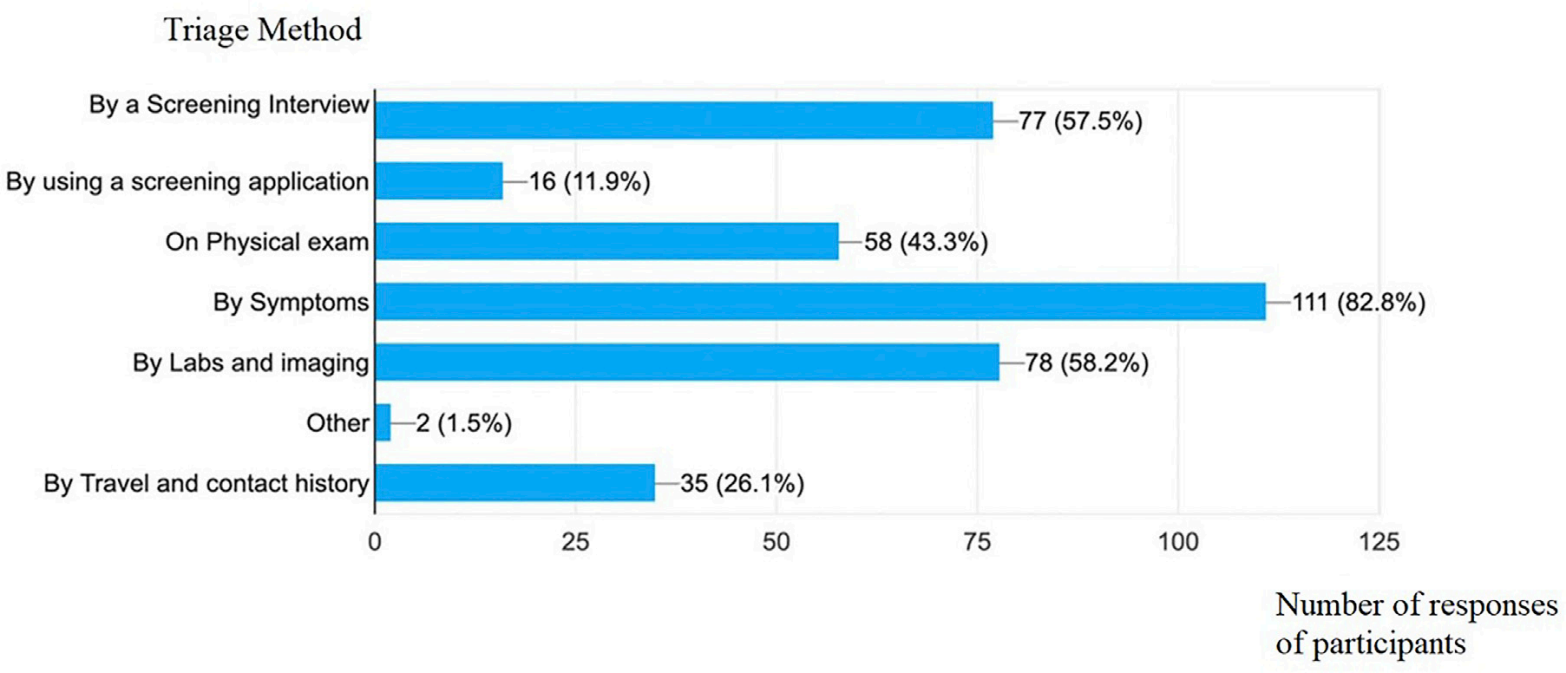
Different screening tools used to identify suspected cases (Lebanon, 2020).

Screening was performed for COVID-19 suspected cases by following one or multiple methods: Symptoms (82.8%), Laboratory testing and imaging (58.2%), Screening interview (57.5%), Physical exam (43.3%), Travel and/or contact history (26.1%), or using a screening application (11.9%) (Figure 2).

Most institutions restructured their EDs to accommodate COVID-19 suspected patients. Some hospitals arranged a new location for triage (57.5%) while others (27.6%) expanded their EDs to directly accommodate all suspected cases within a specific COVID-19 area isolated from the non-COVID patients. Only 8% of the institutions retained their earlier ED space management. Some strategies included isolating each suspected patient within a designated cubicle in the ED (36.8%) while in other instances, patients were asked to keep their masks at all times but were placed in regular ED cubicles (24.6%).

Different combinations of PPEs were applied by institutions: 98% of HCW used masks, gloves, gowns and face shields as minimal protection. Most HCW (48%) used N95, 27% used only a surgical mask, and 25% used both together. The PPE was upgraded by 86% of HCW when the patient needed intubation.

Patients with other acute medical conditions presenting at a moderate to high level of triage were more frequently discharged from the ED, as 67.2% of the HCW reported to discharge them more frequently.

Shortage of staff was mainly seen in Algeria (73%). In case of exposed staff, resuming work was found in Algeria (80% of Algerian respondents), and some European countries (59% of European respondents). Strict visiting restrictions were reported in Algeria, Europe, and Canada. Only in Sweden, a screening application was used to screen suspected cases and for triage management. Other countries used 3 to 4 screening methods: symptoms, travel and contact, screening interview, lab testing and imaging.

The majority of HCW in developed countries reported an architectural change in their Emergency departments. However, such adaptation has not been noted in Algeria (20% of Algerian responses), Lebanon (7% of Lebanese responses) and Ethiopia (100% of Ethiopian responses).

PPE use was variable, mainly in the types of masks. Some HCW in different countries only used surgical mask: Canada (100% of Canadian responses), France (40% of French responses), Ireland (100% of Irish responses), Sweden (25% of Swedish response), United Kingdom (80% of UK responses), and Lebanon (15.5% Lebanese responses).

## Discussion

COVID-19 is a fairly new disease. Hospitals, and specifically EDs, instituted divergent approaches in adapting to the pandemic. Real time PCR tests remain the referential standard for testing in most countries and for organizations like the World Health Organization (WHO) and the Food and Drug Administration (FDA) [19–21]. The survey conducted for this study revealed that the PCR tests with a sensitivity of 63%–78% became available in most hospitals worldwide a few months after the outbreak. The increased rapid availability of the test augmented the testing and identification of cases that were largely asymptomatic or mildly symptomatic [22].

The survey data disclosed that the daily evolving understanding of the disease was reflected in the continuous protocol changes and differing approaches adopted across the globe. Most Healthcare centers recommended sending exposed staff members with a negative PCR home after five to 7 days. In fact, the Centers for Disease Control and Prevention (CDC) instituted a major change in quarantine protocols after the current study’s questionnaire was distributed, recommending that, asymptomatic HCW need no work restrictions with negative PCR results between 5 and 7 days if their vaccinations were up to date. Non vaccinated HCW with negative PCR tests would have to quarantine for 7 days [23]. These instructions indicated that 14 days isolation measures might not be necessary; however, that duration remains the stipulated duration in many countries.

Most surveyed HCW relied primarily on symptoms to identify suspected cases. Also, many institutions in countries evincing high rates of infection began to rely less on travel history because most nations applied travel restrictions and mandatory quarantine periods for travelers, and the virus began to spread locally. However, no studies were conducted on this issue. The CDC reinforced the importance of adequate triage, stipulating a standardized algorithm or questionnaire. However, the questionnaire varied between countries and even between hospitals within the same country. The present study found that the presence of symptoms represents a common and major triage criterion.

Certainly, the COVID-19 pandemic caused ED overcrowding and saturation of hospital beds in zones most prone to the infection, Italy being one of the most affected countries [24]. Multiple strategies were used to isolate suspected patients and decrease the risk of intrahospital propagation to HCW and other patients. Thus, some hospitals created a dedicated space outside the Emergency area for COVID-19 suspected patients, while others limited visitor’ access. The mandate to wear masks inside the hospital premises at all times were also continuously reinforced. Many HCW tended to discharge patients more frequently than before the pandemic, to reduce overcrowding in the emergency areas of the hospitals. However, telemedicine became a crucial solution for non-urgent cases that used to easily overflow EDs at hospitals. This new “emerging star” became possible during lockdown because of the ease of access to teleconferences from home [25].

Perhaps the most important feature discerned by the conducted survey was the increased tendency to discharge moderately sick patients from the ED. Such patients would probably have been admitted to the hospital in the pre COVID-19 times. Additionally, a sharp drop was noted in the emergency flow for non-COVID-19 related presentations because patients were afraid to visit EDs and risk contracting the virus. Two large studies from the United States and Switzerland demonstrated increased morbidity and mortality due to the delay in patients presentation to EDs [26, 27]. Although ED visits decreased dramatically during the pandemic, the acuity of the received cases and the late presentations increased markedly, exposing a dangerous aspect of COVID-19 that could be as threatening as the pandemic itself.

A final outcome of the survey data concerned the use of PPE for examining and intubating suspected patients. In April 2020, the WHO published detailed guidelines on the types of effective PPE [28]. Surgical and N95 masks were found to be equally protective in healthcare settings for non-aerosol-generating care [29]. However, and because of severe PPE shortage in some countries, the authors found an increased risk of exposure to infection when surgical masks were used during intubation [29].

The CDC issued international guidelines for COVID-19 testing, screening tools, emergency room (ER) visits, the wearing of PPE, and other relevant issues related to ER preparedness [23]. The authorities reinforced the rule that patients in the waiting room and the triage must be separated from all patients suffering from acute respiratory infections or any symptom that could indicate a COVID-19. Patients suspected to be infected by COVID-19 should be placed in single rooms with dedicated bathrooms, and their doors always closed. If such accommodations were unavailable, patients who were likely to infected with COVID-19 should wait in a separate, well ventilated area. HCW entering such rooms were asked to wear an N95 mask or equivalent, gowns, gloves, and eye protection. The CDC reinforced virtual visits, instructed visitors on hand hygiene, the use of PPE. In addition, those visiting the hospital could only visit the rooms of their designated patients and were asked to spend the least possible time inside the room. The CDC also recommended restricting the entry of visitors to healthcare facilities, especially if they presented any respiratory symptoms [23].

The importance of this study is the potentially generalizable data about COVID-19 ER preparedness. First, exposed staff members with negative PCR tests after 5–7 days of exposure were allowed to continue to work. Second, the number of visitors to hospitals could be limited. Third, triage should be standardized, and should rely primarily on symptoms. Fourth, patients suspected of being COVID-19 positive should be waiting in a separate area at the medical facility, preferably in well ventilated single rooms. Finally, HCW entering such areas at the medical facilities should wear adequate PPE.

The variability of the results was found between countries and within the same country, reflecting a nonstandard approach for ER COVID-19 management.

### Limitations

This study is an international survey and the distributed questionnaire was in English. Thus, language barriers could present a limitation because many participants were not native users of English.

The different timing of the occurrence and peaks of the pandemic in varied countries presented another drawback. The pandemic was peaking in the United States and Europe when this questionnaire for this study was sent to participants. The incidences of cases climaxed at different times in different parts of the world. The healthcare system responses in different parts of the world were as dynamic as the progression of the disease. Thus, the study could not capture the active issues of the pandemic.

The number of respondents who filled and returned the questionnaire was low. Many HCW were overwhelmed by the COVID-19 circumstances when the questionnaire survey was conducted; many others were quarantined or sick. Numerous HCWs could not access their emails. Such difficulties made data collection difficult. The present study attempted to reach HCW from different countries. This strategy was essential for the aggregation of the maximum possible range of diverse management methodologies for COVID-19 patients.

The present study also tried to contact HCW in China but did not receive any responses from that country. Answers from Chinese HCW could have added value to the study because the current pandemic began in Wuhan and initially spread through China.

### Conclusion

The COVID-19 pandemic had a major impact on EDs worldwide. Protocols and guidelines are rapidly changing as researchers and practitioners continuously attain a better understanding of the virus. Patients with non-COVID-19 related symptoms should be reassured of their safety in visiting EDs before developing complications. Telemedicine could present a viable and beneficial option for such patients. PPE should be used wisely, and a balance must be maintained between HCW safety and PPE shortage in all medical institutions.

It remains to be seen whether technology (telemedicine, artificial intelligence, nanotechnology, and other medical iterations) will become an integral part of emergency medicine worldwide after this pandemic.
