# Supplementary material for: Emergency Department Management of COVID-19 Suspected Patients. An International Perspective
Source: Int J Public Health. 2022 Apr 29;67:1604534. doi: 10.3389/ijph.2022.1604534 (PMC9098677; doi:10.3389/ijph.2022.1604534)
Supplement: Supplementary file 1 [file DataSheet1.DOCX]

**Appendix**

Demographic Data

1. **Age:**

⊗ 18 - 28 ⊗ 29 - 38 ⊗ 39 - 48 ⊗ 49 - 58 ⊗ 59 and above

1. **Gender:**

⊗ Male

⊗ Female

1. **Country of work:**

⊗

1. **Specialty:**

⊗ General Medicine ⊗ Emergency Medicine ⊗ Internal Medicine

⊗ Pulmonary/Critical Care ⊗ Anesthesia ⊗ Other

⊗ Registered Nurse ⊗ Surgical Specialties

1. **Hospital category:**

⊗ University

⊗ Non-University

1. **Type of hospital:**

⊗ Private

⊗ Public

COVID-19 management data

1. **Is COVID-19 PCR available at your institution?**

⊗ Yes

⊗ No.

1. **If Covid-19 PCR is not available, how are suspected patients managed?**

⊗ Suspected patients are sent to another facility for testing

⊗ Samples are taken in our institution, and sent to another facility for testing

1. **Exposed staff management:**

⊗ The exposed staff is sent home for 14 days

⊗ The exposed staff is sent home for 14 days, a PCR test is performed, and once the PCR is negative and the staff is asymptomatic, he/she can come back to work

⊗ The exposed staff continue working with precautions if asymptomatic

⊗ Other

1. **Visitors influx to the Emergency Department:**

⊗ No visitors were allowed

⊗ Visitors should have a face mask on

⊗ Temperature is taken upon entrance

⊗ Limiting visiting hours and number of visitors per patient

⊗Other

1. **Covid-19 suspected cases are recognized:**

⊗ By a Screening Interview (Contact and Travel history)

⊗ By using a screening application

⊗ By Physical exam

⊗ By Labs and imaging

⊗ By Symptoms

⊗ Other

1. **Was the architecture of the Emergency Department changed ? If YES, How?**

⊗ No

⊗ Yes, by creating a triage room outside of the Emergency Department

⊗ Yes, by finding a space for all the suspected cases

⊗ Other

1. **Where are the suspected cases isolated ?**

⊗ Isolation in the Emergency Department while patient have their mask on

⊗ Isolation in a single cubicle

⊗ Isolation with other suspected cases in a designated area of the hospital until results are out

⊗ Other

1. **Non-COVID cases, moderate in severity, were discharged home more frequently:**

⊗ Yes

⊗ No

1. **Critical COVID suspected patients are admitted in:**

⊗ triage area

⊗ main Emergency Department

⊗ transfer to the ICU directly

⊗ Other

1. **Type of PPE s used to examine a suspected patient:**

⊗ Surgical mask

⊗ N95 mask / FFP3

⊗ Gloves

⊗ Double gloves

⊗ Gown

⊗ Shoe Cover

⊗ Head cover

⊗ Face shield

⊗ Goggles

⊗ Other

1. **Do you intubate the COVID suspected patient or you ask for a specialist to do it?**

⊗ Yes, I am intubating the patient

⊗ No, I am calling dedicated personnel (e.g. Inhalation team...)

1. **Type of PPE s used to intubate a COVID suspected patient:**

⊗ Surgical mask

⊗ N95 masks

⊗ Face shield

⊗ Video laryngoscopy

⊗ Plexiglass intubation box

⊗ Transparent plastic cover

⊗ Other
